# Laparoscopic adrenalectomy

**DOI:** 10.4103/0972-9941.19263

**Published:** 2005-10

**Authors:** Nobuo Tsuru, Kazuo Suzuki

**Affiliations:** Department of Urology, Hamamatsu University School of Medicine, Hamamatsu, Japan

**Keywords:** adrenalectomy, aldosteronoma, pheochromocytoma, Cushing's syndrome, adrenal cancer

## Abstract

Laparoscopic adrenalectomy is currently recognized as the gold standard for the treatment of adrenal tumors. In order to assess the current status of laparoscopic adrenalectomy, we reviewed the literature focusing on the indications and contraindications, surgical techniques, complications and new methods. We also reviewed the results separately for aldosteronoma, pheochromocytoma, Cushing's syndrome, and primary or metastatic adrenal cancer.

Laparoscopic adrenalectomy is a safe and effective treatment for adrenal disorders, excluding primary adrenal cancer. There are no differences of the various operative parameters between the transperitoneal and retroperitoneal approaches, so the choice of approach should depend on the surgeon's preference or the patient's circumstances. It is important for the surgeon to remove the tumor and the surrounding fat en bloc, especially in the case of large or irregular tumors because of the potential for malignancy. The surgeon must also immediately switch to an open procedure if the laparoscopic operation becomes difficult.

We conclude that use of laparoscopic adrenalectomy allows the performance of minimally invasive surgery with the advantages of more rapid recovery and a shorter hospital stay than open adrenalectomy.

## INTRODUCTION

Laparoscopic adrenalectomy has become increasingly popular worldwide and is now generally considered to be the standard technique for removal of adrenal tumors.[1,2] In January 1992, laparoscopic adrenalectomy was first performed by Go et al. on a patient with aldosterone-producing adenoma, followed by Suzuki et al. who treated a patient with pheochromocytoma.[3,4] This procedure was first reported by Gagner in 1992.[5] Although it was initially only indicated for small benign tumors, many recent studies have shown that larger, or even metastatic, adrenal tumors are no longer contraindications for laparoscopic adrenalectomy.[6,7] Moreover, the feasibility and efficacy of bilateral or partial adrenalectomy have also been reported.[8,9] The potential benefits of laparoscopic adrenalectomy include less operative blood loss, reduced narcotic requirement for pain relief, a shorter hospital stay, and more rapid recovery.[10]

At present, four different surgical approaches are used for laparoscopic adrenalectomy. These are the anterior transperitoneal approach,[11,12] lateral transperitoneal approach,[13,14] lateral retroperitoneal approach[15,16] and the posterior retroperitoneal approach.[17,18] All of these approaches have various advantages and disadvantages, and good results may be obtained with any of them. Therefore, the approach should be selected according to the surgeon's preference or depending on the patient's circumstances (e.g. the type of disease, tumor size, and patient's wishes).[19]

This article reviews the indications for laparoscopic adrenalectomy, the standard techniques, the results obtained worldwide, and the incidence/management of complications. The current place of laparoscopy in the management of adrenal diseases is also evaluated.

### Indications for laparoscopic surgery

In the early 1990s, laparoscopic adrenalectomy was only used to treat small benign tumors. Since then, the indications have been widened to include various pathological conditions, which are aldosteronoma, pheochromocytoma, Cushing's disease, nonfunctioning adenoma, and other tumors such as myelolipoma and ganglioneuroma. Furthermore, laparoscopic surgery has been performed on bilateral adrenal tumors, large tumors (>5 to 6 cm in diameter), and metastatic adrenal cancer.[7,20,21]

Laparoscopic surgery has been performed on numerous patients in a relatively short period, with the result that conditions such as obesity and previous abdominal surgery are no longer considered to be contraindications.[22] If a patient can withstand anesthesia for open surgery, laparoscopic surgery can also be performed. However, large tumors and symptomatic pheochromocytoma seem to be unsuitable for the retroperitoneoscopic approach because the working space is smaller than with the transperitoneal approach. Moreover, it is better to avoid obese patients or patients with Cushing's disease until the surgeon has sufficient experience with laparoscopic adrenalectomy. Thus, the indications and contraindications for laparoscopic adrenalectomy are closely related to the surgeon's skill and experience. Currently, the only contraindication for laparoscopic adrenalectomy is suspected adrenocortical carcinoma with a relatively large size and invasion of surrounding structures on CT or MRI, because en bloc resection of such tumors together with the ipsilateral kidney and perinephric fat is often required.[23]

### Surgical technique

The transperitoneal route is preferred by many surgeons because of the wider working space and easily visible anatomical landmarks. On the other hand, the retroperitoneal approach provides direct access to the adrenal gland and avoids the risk of intestinal injury or paralytic ileus.

#### Preoperative preparation:

In patients with adrenal tumors of an average size and difficulty (e.g., less than 6 cm in diameter and no invasion), intestinal injury rarely occurs during laparoscopic adrenalectomy. Also, recovery after laparoscopic adrenalectomy is quite rapid, so mild preoperative bowel preparation is sufficient. We usually fast the patient from after the evening meal on the day before the operation and administer a glycerin enema on the morning of surgery. Preoperative control of hormonal imbalance and hypertension in patients with hormone-producing adrenal tumors is the same as for conventional open adrenalectomy.

#### General technique of endoscopic adrenalectomy:

The patient is placed in the flank position (75 to 90 degrees for the transperitoneal approach or the full flank position for the retroperitoneal approach) with the operating table flexed. The surgeon stands in front of (transperitoneal) or behind (retroperitoneal) the patient and uses two ports that are inserted just below the costal margin. The assistant stands on the opposite side and handles the laparoscope and retracting forceps.

**Figure 1:**
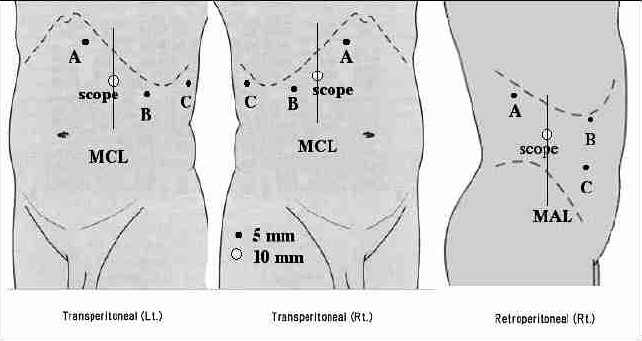
Trocar positions: 10-mm trocar for the laparoscope. A and B: 5-mm trocars for the surgeon. C: 5-mm trocar for the assistant (if necessary). MCL, middle clavicular line. MAL, midaxillary line.

Figure 1 shows the placement of ports for right retroperitoneoscopic adrenalectomy. When an open technique is used (the open method or Hasson's method), a 2–cm skin incision is made at the site of the 10-mm port for the laparoscope. A balloon trocar is inserted through the skin incision and carbon dioxide gas is insufflated to a pressure of 14 mmHg. Subsequently two 5–mm trocars are inserted just below the costal margin and an additional port is inserted, depending on the surgeon's preference. The CO_2_ pressure should then be reduced to 8 to 12 mmHg during the procedure. There is no need for special equipment and standard laparoscopic instruments are sufficient for laparoscopic adrenalectomy.

The retroperitoneal space is filled by the kidney, adrenal gland, and fatty tissue. As the first step, it is very important to localize the adrenal gland within the surrounding fat. If the surgeon attempts to directly approach the adrenal gland at the initial stage of the operation, it is easy to accidentally dissect into the gland. The perinephric fat containing the adrenal gland and kidney should be dissected away from the transversus abdominis muscle (laterally), the diaphragm (above), the psoas muscle (posteriorly), and the pancreas or liver (medially on the left and right sides, respectively) just inside Gerota's fascia. As a result, the adrenal gland will be isolated together with the surrounding fatty tissue.

During separation of the perinephric fat from the diaphragm at the upper pole of the adrenal gland, the superior adrenal vessels are usually exposed and must be carefully clipped and transected. The superior adrenal vessels are sometimes quite large in patients with larger adrenal tumors and must be handled very carefully to avoid massive bleeding. During the course of en bloc dissection, the perinephric fat is divided between the adrenal gland and the upper pole of the kidney using an ultrasonic scalpel. Next, the kidney is reflected downward and the adrenal gland is lifted up by grasping the periadrenal fat. The inferior and posterior adrenal vessels (located below the midline of the adrenal gland) are transected with the ultrasonic scalpel or clipped. The adrenal vein is exposed between the gland and the inferior vena cava (right) or renal vein (left). After 3 to 4 clips have been placed on the adrenal vein, it is transected. Finally, the remaining tissue around the adrenal gland is resected.

An alternative technique for laparoscopic adrenalectomy involves direct exposure of the inferior vena cava (right) or the renal vein (left) and subsequent identification of the adrenal vein as the first step. After transection of the adrenal vein, traction is applied to the cut end of the vessel while the adrenal gland is dissected out. This technique seems to be relatively simple and effective, but early dissection of the adrenal vein may be slightly more dangerous because of the risk of injury to the renal vein or inferior vena cava.

After dissection is complete, the freed adrenal gland is placed in an entrapment sack and retrieved en bloc via the 10-mm port. Next, the trocars are removed under laparoscopic observation. A drain tube is usually not necessary. The fascial incision is closed with 2-0 absorbable sutures at the site of the 10–mm port, while the skin incisions are closed with metal staples.

Figure 1 shows the placement of ports for right as well as left transperitoneal adrenalectomy.

#### Transperitoneal approach:

Three or four trocars are inserted along the costal arch. In patients undergoing left adrenalectomy, the peritoneum is incised at the line of Toldt along the lateral border of the spleen until the greater curvature of the stomach can be visualized. The left adrenal gland can be easily approached after the spleen is reflected medially and falls away under its own weight. Whether surgery is being done on the left or right side, the adrenal gland is separated from the surrounding tissues. After dividing the kidney from the adrenal gland, the left adrenal vein (which enters the renal vein) is identified. The adrenal vein is then dissected free, clipped, and transected.

In patients undergoing right adrenalectomy, the peritoneum is incised along the liver as far as the diaphragm so that the right lobe of the liver falls away medially. After the inferior vena cava is visualized, dissection proceeds upwards along the lateral margin of this vessel until the main adrenal vein is exposed. This is carefully dissected, clipped, and transected. After that, the adrenal gland is freed and removed.[24]

#### Retroperitoneal approach:

A 2-cm skin incision is made above the iliac crest on the midaxillary line and the posterior perirenal space is developed with the index finger. The space is expanded using a balloon dilator placed just outside Gerota's fascia. A 5-mm trocar is inserted below the costal margin on the posterior axillary line by the finger-guided method and a balloon trocar is inserted through the skin incision. After the peritoneal membrane has been reflected medially, one or two 5-mm trocars are inserted on the anterior axillary line under endoscopic observation.

When performing this operation, it is very important to create a wide enough space for surgery. To improve visualization of the operative field, the abundant fatty tissue in the posterior pararenal space (sometimes called the “flank pad”) should be removed. Gerota's fascia and the lateroconal fascia are incised vertically. The incision is extended upwards near the diaphragm so as to make a wide opening. The adrenal gland and kidney should be dissected away from the perinephric fat, after which the fat floats freely on the surrounding tissues as an ovoid mass. Between the inferior vena cava (right) or the renal vein (left) and the adrenal gland, the adrenal vein is exposed, after which it is clipped and transected.

### Worldwide results

#### Aldosteronoma:

Primary hyperaldosteronism presents with hypertension and hypokalemia due to autonomous hypersecretion of aldosterone from the adrenal cortex. Approximately 60–75% of patients with primary aldosteronism have a unilateral aldosterone-producing adenoma (APA), and this is generally called Conn's syndrome. Idiopathic hyperaldosteronism (IHA), occasionally associated with bilateral adrenal hyperplasia, occurs in about 25% of the patients with primary aldosteronism. It is essential to separate IHA from APA, because adrenalectomy rarely cures hypertension in patients with the former condition.[25] In contrast, surgical resection of an aldosteronoma commonly results in the elimination of hypertension, although some patients still require medication.

Brunt et al. reported that hypertension improved in 92% of patients after laparoscopic adrenalectomy, but 69% of them still required medication.[26] Generally, the risk factors for persistent hypertension are the presence of hypertension on discharge from hospital, the response to spironolactone, a long preoperative duration of hypertension, and a high preoperative plasma renin activity. Harris et al. reported that advanced age was associated with a significantly lower cure rate.[27] On the other hand, Rossi et al. reported that the duration of hypertension before surgery was the main risk factor for persistent hypertension.[28]

Regarding the surgical management of APA, it has been controversial as to whether it is more appropriate to perform total adrenalectomy or partial resection. Some studies have demonstrated that partial resection or enucleation of an APA is preferable to unilateral adrenalectomy.[29] However, a second functioning microadenoma may remain in the ipsilateral adrenal gland after partial adrenalectomy, leading to persistence of hypertension and hyperaldosteronism. Accordingly, total adrenalectomy is preferable in patients with APA.[30]

We reviewed 5 articles focusing on laparoscopic adrenalectomy in a total of 170 aldosteronoma patients (Table 1). The mean operating time, estimated blood loss, and hospital stay were 175 min, 45 m, and 2.7 days, respectively. The mean tumor diameter was 2 cm and the conversion rate to open surgery was 4.1%. Elimination of hypertension was only achieved in 55% of the patients, although almost all of them improved to some extent.[26,27,28,30,31]

**Table 1:** Laparoscopic adrenalectomy for aldosteronoma

References	No. Pts.	Mean Age	Approach (No.)	Mean Ope. Time (mins.)	EBL (cc)	No. Comversion	No. Complications	Hospital stay (days)	No. Cures Hypertension	Tumor size (cm.)
Brunt et al.[26]	29	48	Transperitoneal	148	60	0	6 (20%)	2.2	8/26 (31%) (improved; 92%)	1.9
Harris et al.[27]	21	48	Transperitoneal	158		1.92 (9.5%)	0	3.1	13/21 (62%·j	
Rossi et al.[28]	30	51.2	Transperitoneal	183		1 (3.3%)	2 (6.7%)	2.2	20/30 (67%) (improved; 97%	2
Ishidoya et al.[30]	63	50.2	Transperitoneal (21)							
			Retroperitoneal (42)	199	57.3	0			improved; All	1.6
Kalady et al.[31]	27	47	Transperitoneal	153	88	4 (14.8%)	2 (7.4%)	3.6		3.3
Overall	170	49.2	Transperitoneal (128)							
			Retroperitoneal (42)	175	45	7 (4.1%)	10/107 (9.3%)	2.7	42/77 (54.5%) (improved; 98%)	2

#### Pheochromocytoma:

Pheochromocytoma is a neuroendocrine tumor that secretes large amounts of catecholamines, causing hypertension, episodic tachycardia, hypovolemia due to severe vasoconstriction, and adrenegenic cardiomyopathy. Among patients with pheochromocytoma, 10% have multiple endocrine neoplasia (MEN) type 2A or 2B, with bilateral adrenal tumors, extra-adrenal tumor(s), and histological malignancy. Surgery for pheochromocytoma is associated with an increased operative risk due to special problems with hemodynamics and cardiovascular complications. Use of laparoscopic surgery to treat pheochromocytoma was once controversial, because it was believed that an increase of intra-abdominal pressure during the operation would lead to hemodynamic changes and provoke the additional release of catecholamines. However, Fernandez-Cruz reported that laparoscopic surgery for pheochromocytoma was associated with a smaller increase of the catecholamine level than open surgery, and that episodes of hypertension were related to direct manipulation of the tumor.[32] Although every effort should be made to ligate the adrenal vein before starting dissection of the gland, this is not always strictly necessary. Despite early ligation of the adrenal vein, release of catecholamines may occasionally occur during tumor manipulation.[33]

A review of the recent literature on laparoscopic adrenalectomy for pheochromocytoma collected 7 studies reporting on 121 patients (Table 2). A transperitoneal approach was used in all but two cases. The mean operating time was 3 hours, mean blood loss was 160 mL, and mean tumor diameter was 4.8 cm. The mean hospital stay was 4 days and complications developed in 13.7% of the patients. Seven patients (6.7%) needed conversion to open surgery.[26,31,33–37]

**Table 2:** Laparosacopic adrenalectomy for pheochromocytoma

References	No. Pts.	Mean Age	Approach (No.)	Mean Ope. Time (mins.)	EBL (cc)	No. Comversion	No. Complications	Hospital stay (days)	Tumor size (cm.)
Brunt et al.[26]	35	42	Transperitoneal	208	147	2 (5.7%)	7 (22.9%)	3.4	3.4
Kazaryan et al.[34]	9	48	Transperitoneal	132	178	0	0	3.2	6.4
Kim et al.[35]	15	45.2	Transperitoneal	171	189.5	0	0	5.6	5.2
Flavio Rocha et al.[33]	12	54	Transperitoneal (11)						
			Retroperitoneal (2)	127	105		0	4.18	4.4
Kalady et al.[31]	28	53	Transperitoneal	181	150	3 (10.7%)	3 (10.7%)	3.7	5.2
Bentrem et al.[36]	4	43	Transperitoneal	218	188			3	4.3
Thomson et al.[37]	18	48	Transperitoneal	180 (unilateral)				
				450 (bilateral)	200	2 (10.5%)	6 (33.3%)	5	6
Overall	121	47.5	Transperitoneal (119)						
			Retroperitoneal (2)	180	160	7/105 (6.7%)	16/117 (13.7%)	4	4.8

#### Cushing's syndrome:

Cushing's syndrome may be ACTH-independent or ACTH-dependent, with the former type being caused by adrenocortical adenoma, carcinoma, or hyperplasia, while the latter is often due to a pituitary adenoma that causes bilateral adrenal hyperplasia (Cushing's disease). Conventional open adrenalectomy requires a large incision to gain access to a relatively small gland, and this is a serious problem in patients with Cushing's syndrome. Since laparoscopic surgery requires a smaller skin incision, it may decrease the risk of poor wound healing. Nevertheless, the performance of laparoscopic adrenalectomy for Cushing's syndrome is hampered by the presence of abundant retroperitoneal fat, which seems to be a feature of this condition.[1]

Extending the use of laparoscopy to the performance of bilateral laparoscopic adrenalectomy is a reasonable alternative for the treatment of ACTH-dependent Cushing's syndrome after failure of surgery for a pituitary adenoma (Cushing's disease) or when the source of ACTH production cannot be resected or localized.[38]

Gill reviewed 6 studies on laparoscopic surgery for Cushing's syndrome that included 64 patients.[39] The mean operating time was 2.5 hours for unilateral adrenalectomy and 5.2 hours for bilateral surgery. Estimated blood loss was 177 mL for unilateral surgery and 298 mL for bilateral procedures. Conversion to open surgery was done electively in 5 cases due to difficulty with dissection or severe hypercapnia. Complications developed in 6 patients (9.5%), and 1 patient died of gastrointestional bleeding 2 weeks after surgery.

#### Primary or metastatic adrenal malignancies:

Primary carcinoma of the adrenal cortex is a rare endocrine neoplasm with a worldwide incidence of approximately two per million persons. Complete surgical resection is the only potentially curative treatment for this tumor.[40] It is not always easy to make a correct preoperative diagnosis, but it is generally considered that adrenal tumors with a diameter of 6 cm or more are probably malignant.[41] Presently, laparoscopic surgery is not indicated for patients with tumor invasion of adjacent organs.[2] Dackiw et al[42] reported 2 cases of adrenal cortical carcinoma that recurred 6 months after surgery. We have also experienced a patient with Cushing's syndrome due to cancer of the left adrenal cortex who developed intraperitoneal dissemination at 19 months after laparoscopic adrenalectomy.[43] If there is any difficulty with performing dissection due to invasion of the surrounding tissues or a large tumor size, the possibility of malignancy should be recognized and conversion to open surgery should be done without hesitation.

Use of laparoscopic adrenalectomy for metastatic adrenal cancer has been reported recently.[44,45] When the primary tumor has been controlled and there are no other metastatic lesions, complete resection of an adrenal metastasis will almost always prolong survival and therefore laparoscopic resection is useful for the management of metastatic adrenal cancer. However, it is not indicated when preoperative imaging shows possible tumor infiltration of the surrounding tissues.

Miccoli et al. evaluated 22 patients who underwent laparoscopic adrenalectomy for suspected metastatic adrenal cancer and were successful in all but three cases. Among 13 patients (excluding those histologically found to have an adenoma), local relapse only occurred in one case and 8 patients were alive and disease-free after a mean follow-up period of 39 months.[7] Thus, laparoscopic radical resection offers a much better postoperative outcome for patients with metastatic adrenal tumors.

#### New techniques:

Since its introduction, laparoscopic adrenalectomy has already come to be considered as the gold standard for adrenal surgery. However, there are ongoing developments of this surgical procedure, including the use of new instruments or ablation methods. Robot-assisted adrenalectomy has been attempted, but Morino et al. concluded that it was inferior to standard laparoscopic surgery in term of feasibility, morbidity, and cost.[46]

Cryosurgery and radiofrequency ablation of adrenal lesions have been described as less invasive and adrenal-sparing procedures.[47]

Outpatient adrenalectomy has also been performed at several institutions. Although it is feasible and safe with a satisfactory clinical outcome, ambulatory adrenalectomy should be restricted to carefully selected patients (e.g. younger, lower body mass index, small tumor, and no pheochromocytoma) and requires a highly experienced surgeon.[48]

### Complications and their management

The most serious intraoperative complications are vascular damage and organ injury. Bleeding is the most important cause of conversion to open adrenalectomy but this should become less common as a surgeon's experience increases. When blind dissection of the fatty tissue is done to detect an adrenal tumor, there is a high risk of injuring large vessels (superior adrenal vessels, adrenal vein, renal artery and vein, inferior vena cava, etc.) or any of the organs in that region (adrenal gland, kidney, liver, pancreas, etc.).

In a retrospective meta-analysis, Brunt et al[49] evaluated the complications of 1,633 adrenalectomies reported in 50 studies. Bleeding occurred in 71 patients (4.7%) and required transfusion in 27 patients (1.8%). Bleeding that required conversion to open surgery occurred in 1.6% of the patients, accounting for 30% of all conversions. Pneumothorax can be induced by injury to the diaphragm. Dissection of the upper pole of the adrenal gland should be done with elevation of the surrounding fat in order to avoid direct contact of the electrocautery probe or ultrasonic scalpel with the diaphragm.

Postoperatively, the main advantages of laparoscopic adrenalectomy are fewer pulmonary complications and a dramatic decrease of wound complications.[50] Postoperative wound and pulmonary complications were reported in 1.5% and 0.6% of patients respectively, with both being much less frequent than after open adrenalectomy (*p*=0.0001).[49] Pulmonary embolism secondary to deep venous thrombosis is a serious complication. Although routine use of heparin is controversial, mechanical intermittent compression devices for the calves are recommended during surgery.

## CONCLUSIONS

Laparoscopic adrenalectomy should be considered as the surgical treatment of choice for most adrenal neoplasms, with primary carcinoma of the adrenal cortex being the only absolute contraindication at present. Large and/or irregular adrenal tumors have a high risk of malignancy and require radical en bloc resection to avoid local recurrence. It is therefore important to investigate tumors carefully by preoperative imaging. The success or failure of laparoscopic adrenalectomy depends on tumor characteristics such as the presence of adhesions or strong connections to other organs rather than on any specific histology. A highly skilled and experienced surgeon can safely perform laparoscopic adrenalectomy in patients with pheochromocytoma or patients with large adrenal tumors.

To avoid an adverse outcome, the surgeon must not hesitate to convert a patient to open surgery when required, and conversion should not be considered as a “failure”. The advantages of laparoscopic adrenalectomy include fewer postoperative wound and pulmonary complications, allowing more rapid recovery and a shorter hospital stay than after open adrenalectomy. However, further studies and a longer follow-up period are needed to more precisely assess the value of laparoscopic adrenalectomy.
